# Advancing Virtual Bioequivalence for Orally Administered Drug Products: Methodology, Real-World Applications and Future Outlook

**DOI:** 10.3390/ph17070876

**Published:** 2024-07-03

**Authors:** Sivacharan Kollipara, Frederico Severino Martins, Rebeka Jereb, Dejan Krajcar, Tausif Ahmed

**Affiliations:** 1 Biopharmaceutics Group, Global Clinical Management, Dr. Reddy’s Laboratories Ltd., Integrated Product Development Organization (IPDO), Bachupally, Medchal Malkajgiri District, Hyderabad 500 090, Telangana, India; sivacharankollipara@drreddys.com; 2 Simulations Plus, Inc., Lancaster, CA 93534, USA; frederico.martins@simulations-plus.com; 3 Clinical Pharmacology and Modeling & Simulation, Sandoz Global Development, Lek d. d., Verovškova ulica 57, SI-1526 Ljubljana, Slovenia; rebeka.jereb@sandoz.com (R.J.); dejan.krajcar@sandoz.com (D.K.)

**Keywords:** GastroPlus, PBBM, regulatory, variability, virtual bioequivalence

## Abstract

Bioequivalence studies are pivotal in generic drug development wherein therapeutic equivalence is provided with an innovator product. However, bioequivalence studies represent significant complexities due to the interplay of multiple factors related to drug, formulation, physiology, and pharmacokinetics. Approaches such as physiologically based biopharmaceutics modeling (PBBM) can enable virtual bioequivalence (VBE) assessment through appropriately developed and validated models. Such models are now being extensively used for bioequivalence risk assessment, internal decision-making, and the evaluation of drug and formulation factors related to bioequivalence. Depiction of the above-mentioned factors through the incorporation of variability and development of a virtual population for bioequivalence assessment is of paramount importance in utilizing such models. In this manuscript, we have portrayed our current understanding of VBE. A detailed explanation was provided with respect to study designs, *in vivo* variability, and the impact of physiological, drug, and formulation factors on the development of the population for VBE. Furthermore, strategies are suggested to incorporate variability in GastroPlus with an emphasis on intra-subject and inter-occasion variability. Two industrial case studies pertaining to immediate and modified release formulation were portrayed wherein VBE was utilized for decision-making and regulatory justification. Finally, regulatory understanding in the area of VBE, along with future perspectives, was detailed.

## 1. Introduction

In the dynamic landscape of generic drug development, bioequivalence studies stand as a pivotal component, ensuring therapeutic equivalence between a generic product and its innovator counterpart. However, the intricate interplay of physiological variables, pharmacokinetics, and formulation characteristics presents a multifaceted challenge in achieving bioequivalence. It is in this complex setting that Physiologically Based Biopharmaceutics Modeling (PBBM) has risen as a transformative force. The integration of PBBM into the development pipeline has revolutionized our approach, offering a sophisticated platform for strategic decision-making, thus minimizing the reliance on resource-intensive *in vivo* studies [1].

This review manuscript delves into the profound impact of PBBM in streamlining generic drug development—a narrative of innovation underpinned by rigorous science. PBBM has been pivotal in refining formulation selection, expediting biowaivers, elucidating f2 mismatch conundrums, assessing the clinical relevance of drug-drug interactions (DDIs), and crafting clinically relevant dissolution specifications (CRDS) [2,3,4,5,6,7]. These areas, critical to the assurance of bioequivalence, have been redefined by the analytical prowess of PBBM, ushering in a new era of efficiency and precision [8].

Bioequivalence is a complex paradigm impacted by a mosaic of different variables—study designs, the intrinsic variability of formulation product, physiological considerations, and the quintessential ADME (absorption, dissolution, metabolism, and elimination) processes. When addressing a healthy or patient population, the nuances of these factors become increasingly pronounced. It is the thorough understanding and accurate simulation of these attributes in virtual populations that PBBM aims to master, setting a foundation for *in vivo* mechanistic predictions. In the subsequent sections, we unravel the intricacies of these considerations and their integration into the development of a virtual population for PBBM applications [1,2].

By dissecting the multifactorial determinants of bioequivalence and illustrating the application of PBBM, this manuscript aims to provide a comprehensive review of how to best use this modeling approach by not just enhancing our current capabilities but also charting the course of generic drug development in the future. Accounting correctly for variability from all contributing parameters is an important part of the development of a virtual population that could capture *in vivo* variability. The following sections cover the considerations of these factors during the development of a virtual population [1,2].

## 2. Study Designs

Bioequivalence studies play a crucial role in pharmaceutical research, acting as a cornerstone in the regulatory approval of generic drugs. Although biopharmaceutics classification system (BCS)-based biowaiver approach allows waiving bioequivalence studies for BCS Class I and III drugs, conducting the bioequivalence studies is inevitable for other classes of drugs as well as for those that are not meeting BCS-based biowaivers criteria. These studies verify that the generic drug mirrors the pharmacokinetic behavior of its brand-name counterpart. To ensure the reliability and accuracy of bioequivalence assessments, it is vital to choose the right study design [3].

Both the generic and innovator formulations should be administered under identical conditions: the same dose, active pharmaceutical ingredient (API), and under the same physiological conditions (e.g., fasted, fed). They can be given as single or multiple doses. However, a single dose is typically preferred for bioequivalence evaluations for immediate-release (IR) products. This is because single-dose studies are more adept at gauging the drug’s release rate and the extent of its entry into the systemic circulation compared to steady-state studies [4]. However, for formulations with different release rates (e.g., modified release (MR) dosage forms), multiple studies are required that consist of steady-state studies, food effect, dose proportionality, and additional strength biowaivers (in case dissolution criteria or formulation proportionality criteria does not meet the requirements).

The selection of a bioequivalence study design is influenced by several factors, including the drug’s physicochemical properties, its pharmacokinetics, and the variability in drug exposure. The two prevalent designs are parallel and crossover. In parallel studies, participants are randomly assigned to one of two groups, receiving either the test drug (T) or the reference drug (R) throughout the study [5]. In contrast, crossover studies ensure that each participant receives both drugs in successive phases. This design is favored in standard bioequivalence studies because it accounts for intra-individual variability in drug disposition, therefore enhancing statistical power [6].

However, crossover studies come with the challenge of potential carryover effects. This means the drug given in the initial phase might influence the response to the drug administered in the subsequent phase. To counteract this, a washout period is typically introduced between the two phases, allowing participants to revert to baseline conditions [7,8,9].

Bioequivalence studies can be either nonreplicated or replicated. The standard format is a two-period, two-formulation, two-sequence crossover study, which uses a nonreplicated design. For most drugs, an average bioequivalence approach suffices for statistical analysis. Yet, for drugs deemed “highly variable” (where the intra-subject coefficient of variation (intra-CV) exceeds 30% for peak drug levels (C_max_) or the area under the plasma drug concentration-time graph (AUC)), a replicated design provides a more accurate estimation of individual responses. Such replicate design can be partial replicate (i.e., reference replicate) or full replicate (i.e., both reference and test replicate). Therefore, the choice of study design hinges on factors like drug variability and the imperative for precise bioequivalence assessments [6,8,10].

Determining the appropriate sample size and its power is another pivotal aspect of designing a bioequivalence study. In statistical terms, power denotes the likelihood of dismissing the null hypothesis when the alternative hypothesis holds true. Given that the alternative hypothesis in these studies posits bioequivalence, the study’s power equates to the chance of affirming bioequivalence when the products are genuinely bioequivalent [6,11,12,13]. Ascertaining the optimal sample size is crucial since it guarantees sufficient power, making this decision a cornerstone in bioequivalence study design. Oversized samples not only escalate study expenses but also expose numerous subjects to the drug unnecessarily. Conversely, undersized samples heighten the risk of type 2 errors, potentially leading to study shortcomings. As per the statistical protocols set by the U.S. FDA and EMA, bioequivalence studies should aim for 80% or 90% power [4,9,14]. To decide on the sample size, one must estimate the variability of primary pharmacokinetic metrics. The sample size is, at the same time, guided by the expected test-to-reference ratio, the study design, type I error, and target power.

Other factors to consider in the study design are population, dose selection, and type of studies (i.e., fasting or fed). Typically, the bioequivalence and product-specific guidance of the U.S. FDA and EMA do not specify a particular ethnic population in which the bioequivalence studies should be conducted. Hence, the generic company may conduct bioequivalence studies on any local population. While performing the VBE, this population can be considered in the software. From a dose-selection perspective, the highest strength is generally used in the bioequivalence study. However, lower doses can also be selected in case of pharmacokinetic non-linearity or toxic effects at higher concentrations. In general, the U.S. FDA recommends studies in both fasting and fed conditions; however, EMA indicates study requirements as per label condition (e.g., with or without food). These administration conditions must be appropriately considered while performing VBE simulations.

## 3. Variability and Its Impact on VBE Studies

In recent times, variability in VBE has gained prominence. For VBE findings to be universally recognized and credible, it is paramount to seamlessly and accurately incorporate relevant variability into physiologically based pharmacokinetic (PBPK) models in a mechanistic manner. Delving into pharmacokinetic measurements, such as AUC or C_max_, two layers of variability emerge—within-subject and between-subject variability (BSV) [15,16,17]. Within-subject variability (WSV), also known as intra-subject variability (ISV) or inter-occasion variability (IOV), is inherent to individual subjects, manifesting when the same person is assessed multiple times. Even with consistent drug administration, a subject’s bioavailability measurements can oscillate over distinct time intervals as natural fluctuations in an individual’s physiological parameters over time. These can be influenced by factors such as circadian rhythms, changes in diet, physical activity, fluctuations in drug dissolution, gastric pH and emptying first-pass effect, and transient health conditions. Such fluctuations might arise from minor variations in drug administration or even discrepancies in the drug product’s manufacturing process, like intra-lot differences. Essentially, this variability encapsulates the diverse responses a single individual might display over time [6,18,19]. On the other hand, BSV or inter-subject variability surfaces when comparing bioavailability measurements across a study’s participants. Given the unique physiological attributes, genetic factors, and metabolic processes of each individual, a group administered the same drug under identical conditions might still present varied bioavailability profiles. For instance, one person’s rapid drug absorption might starkly contrast with another’s slower rate. Collating these individual measurements forms a distribution for the entire study group, with the mean denoting the collective average bioavailability and the variance shedding light on the range of observed responses. In essence, while ISV zeroes in on the range of outcomes from a singular individual over repeated instances, BSV underscores the differential responses across a study’s participants [18,20,21,22].

ISV plays a pivotal role in the precision of bioequivalence evaluations for crossover designs. It shapes the 90% confidence intervals for the average differences in the logarithmically transformed pharmacokinetic markers, such as C_max_ and AUC. These metrics are vital in comparing the test and reference products. When the intra-CV of C_max_ and/or AUC surpasses 30%, the required number of participants for validating bioequivalence using the 90% confidence interval surges considerably, often rendering such studies unfeasible [23].

## 4. Variability of Physiological Factors

The gastrointestinal (GI) tract, characterized by its complex physiology, is instrumental in the absorption of orally administered drugs. This system is central for determining drug bioavailability, directly impacting bioequivalence outcomes. Variabilities inherent to the GI tract, such as gastric emptying rates, intestinal motility, and pH gradients, can shape drug dissolution and absorption. For instance, the mean residence time (MRT) in regions like the stomach, small intestine, and colon can influence how long a drug remains in a specific area, affecting its dissolution and absorption kinetics.

The pH variations across the GI tract, from the stomach’s acidic environment to the intestines’ slightly alkaline setting, can alter the ionization state of drugs. This is particularly true for weak acids or bases, which can impact their solubility and permeability, thus resulting in diverse absorption profiles. The bile salt concentrations play a significant role in the solubilization of lipophilic compounds, especially in regions like the jejunum.

Based on magnetic resonance imaging measurement of gastric emptying time, it takes about 45 min to return to fasted gastric volumes after consuming a 240 mL glass of water. However, after a standardized high-caloric breakfast as defined by the U.S. FDA, it takes over 6 h. Small-intestinal transit times consistently range between 4.3 and 4.6 h. By contrast, colonic transit times vary significantly, ranging from approximately 18 to 34.2 h. The volume of fluids in the GI tract plays a role in determining drug concentrations within the GI tract, influencing both dissolution behavior and the permeation driving force. In a fasted state, resting gastric fluid volumes are reported to fluctuate between 25 and 45 mL, showing a noticeable variation among individuals. On the other hand, fluid volume in the fed state is contingent upon the amount of food consumed and the elapsed time post-ingestion. In the small intestine during a fasted state, fluid volumes average around 43 ± 14 mL, with values ranging from 5 to 159 mL. This fluid is not uniformly distributed but is present in sporadic pockets. In the ascending colon, average fluid volumes in the fasted state fall between 7 and 22.3 mL, while in the fed state, it is about 29.9 mL [24,25]. Tanaka et al.’s research [26] highlighted the role of luminal fluid volume in the GI tract on drug absorption. Their findings emphasized a pronounced absorption effect on a class 3 drug (atenolol) in comparison to a class 1 drug (metoprolol).

An evaluation of relationships between ISV and BSV for drugs in each BCS class revealed that class III drugs exhibited a pronounced ISV. Since the absorption of class III drugs was rate-limited by the permeability, this result might be explained by an increased ISV in absorption rate due to the poor membrane permeability of drugs. This is possibly due to factors such as low permeability, as previously highlighted by Yamashita and Tachiki [27].

Masahisa Sugihara [28] underscores the role of metabolic enzymes like CYP3A4 and CYP2D6. For instance, results demonstrated high ISV for drugs metabolized by CYP3A4, while those showing a high BSV are substrates of CYP2D6. Further analysis revealed that the affinity of enzyme (K_m_) of CYP3A4 can be a parameter that can have significant impact on ISV for AUC parameter.

In the realm of VBE, the nuances of GI physiology are paramount. These virtual evaluations utilize sophisticated modeling techniques to predict drug formulation behavior without exhaustive *in vivo* trials. However, to ensure accuracy, models must encapsulate the diverse physiological factors influencing drug absorption. By considering these variabilities, these models can offer realistic predictions of drug behavior. Overlooking such intricacies might jeopardize patient safety and therapeutic outcomes. Hence, integrating GI physiological variabilities is essential for the credibility and success of VBE assessments. However, during the actual execution of the study, a few intercurrent events, such as vomiting or diarrhea, may happen to subjects involved in the study. These are considered to be adverse events and will be reported in the study report. The inclusion of subjects with such adverse events depends on the pre-defined criteria in the study protocol. For example, if vomiting occurs within 1 h of administration, there is the possibility that the dosage form is expelled, and thus, the subject may not be included in the study outcome analysis. However, considerations of vomiting and diarrhea may not be possible in the simulations at this point.

## 5. Variability of API and Formulation

Some API and formulation properties, such as low solubility, low permeability, absorption window in the GI tract, variable dissolution rate, GI degradation, first-pass metabolism, or intestinal transport, can greatly affect BSV and ISV.

For example, Davit et al. [29] reviewed 1010 BE studies of 180 different drugs evaluated by the FDA’s Office of Generic Drugs during 2003–2005, of which 31% had highly variable C_max_ or AUC. About 60% of the highly variable drugs were highly variable due to API characteristics, such as low aqueous solubility, acid lability, low oral BA, and extensive first-pass metabolism. Formulation performance contributed to the high variability for about 20% of the highly variable drugs.

Similarly, Sato et al. [30] showed a negative relationship between absolute oral BA and ISV of PK exposure (C_max_ and AUC) following oral administration of IR drugs under fasted conditions. Drugs with a poor absolute oral BA of less than 5% showed high intra-CV in the range of 30–65%. In contrast, drugs with high absolute oral BA of more than 80% showed low intra-CV of less than 20%. They also showed that acidic drugs with pKa < 6 had higher intra-CV of C_max_ than AUC compared to other types of drugs.

Sugihara et al. [28] showed that ISV of BCS class III drugs increases in association with a decrease in drug permeability. Since the absorption of BCS class III drugs is rate-limited by the permeability, it was suggested that, for such drugs, the low Peff might be a risk factor to cause a large ISV. They also found that BCS Class II drugs with a solubility-limited absorption showed high ISV. Thus, low permeability, solubility-limited absorption, and high affinity to CYP3A4 were identified as risk factors for high ISV in oral drug absorption.

API and formulation can have significant importance on the variability of the virtual population. From the API perspective, its form, solubility vs. pH profile, precipitation potential, particle size, and regional permeability of the drug in the GIT can be a few factors that are to be considered. For weakly basic drugs, the solubility vs. pH profile decreases steeply from acidic to basic conditions. Hence, depending on BSV in GI pH, different extents of precipitation can happen, contributing to absorption variability. Regional permeability, arising from specific transporter expression or pH, can be another contributing factor to the variability. A PBPK or PBBM, which has been developed considering pH-dependent solubility profile and/or transporter expression, can have the potential to perform parametric sensitivity analysis and thus can help to understand sources of variability. Furthermore, mixtures of API and particle size are also factors to be considered in formulation variability.

From a formulation perspective, aspects related to dosage forms such as IR or MR are to be considered. Within IR dosage forms, factors related to disintegration and dissolution can play a role in the variability of the product. With regard to dissolution, depending on the individual pH, fluid volumes, and surface area, the dissolution rate can vary within individuals and thus contribute to the variability. Delayed release (DR) formulations can exhibit different types of variability as compared to IR formulations due to enteric coating. DR formulations have an enteric coating that dissolves at specific pH conditions. BSV in pH conditions can contribute significantly to the time needed for coating dissolution, resulting in variable T_max_, C_max_, and AUC. Additionally, once the enteric coating dissolves, the aspects related to variability shall be similar to those of the IR formulations. Extended-release (ER) formulations represent more complex formulations than IR and DR formulations. Many factors can lead to the variability of ER formulations, such as the site of release, absorption, dissolution mechanism release rate, etc. Coupled with these factors, when ER formulations are manufactured with low-solubility drugs exhibiting pH-dependent solubility, their variability is governed by many factors, and optimization of the virtual population becomes much more complex. Additionally, for all formulations, depending on generic formulation, composition, and manufacturing process similarity with that of the reference product, different variability can be anticipated for reference and generic formulation, which needs to be considered accordingly. Since formulation-related parameters impact absorption-related aspects, it becomes important to consider these parameters when addressing variability during the absorption phase. In addition to parameters such as permeability, the variability of *in vivo* dissolution parameters can be altered systematically to disentangle these two sources of absorption variability.

## 6. Considerations of Compartmental vs. Full PBPK Model

While API dissolution and absorption are described by a mechanistic model, the disposition can be described by a compartmental model or a full PBPK model. For generic applications, a simple compartmental model is usually sufficient (i.e., 1, 2, or 3-compartmental models). The mean parameters of 1-, 2-, or 3-compartmental models (clearance (CL), volume of distribution (V_d_), distribution rate constants) can be extracted from mean plasma concentration profiles after intravenous (IV) administration of API, and decision on an appropriate number of compartments can be made based on Akaike Information Criterion (AIC) [31,32]. Another option is to use parameters determined with population pharmacokinetic (popPK) modeling of individual data after IV administration [33]. When simulating a virtual population, variability in disposition parameters must be included. Such a virtual population is designed to match the actual population with respect to age, gender, race, weight range, and disease conditions. Default GastroPlus (a mechanistically based simulation software that can simulate plasma concentration-time profiles of various routes of administration) %CV parameters for CL, V_d_, and rate constants are 40%, 20%, and 20%, respectively. One could optimize these values based on popPK analysis performed on the specific study and use %CV of CL and V_d_ from popPK analysis [34]. The %CV could also be optimized based on a comparison of simulated plasma concentration profiles and mean and range of PK parameters with the ones observed in the *in vivo* study [31,32]. Other software, such as SimCyp and PKSIM, also possess similar options for the inclusion of variability for the population simulations.

On other occasions, a more complex full PBPK model must be implemented. For example, Franco et al. [35] first developed a compartmental PK model for ketoconazole and, after gaining confidence in the absorption-related parameters, applied the full PBPK model. Using the full PBPK model, the drug substance disposition is characterized by tissue-plasma partition coefficients (K_p_), which dictate the steady-state volume of distribution (V_ss_). Elimination can be characterized by the sum of renal, blood, and hepatic clearance. Hepatic clearance can be metabolism and transport-related and can be described by the Michaelis–Menten constant (K_m_) and maximum metabolic rate (V_max_) for specified enzymes and/or transporters. It may be challenging to obtain the estimates for Km and Vmax values and their %CV. However, it is even more challenging to know which value to use for a simulation of a virtual population with varying K_m_ and V_max_ values. There is an option to optimize %CV based on a comparison of simulated plasma concentration profiles and PK parameters with the observed ones from the *in vivo* study by trying to match observed variability (individual profiles). Also, parameter-sensitivity analyses can be helpful. However, the isolation and identification of ISV from BSV and drug variability from system variability are key to avoiding over-parameterization of PBBM [36].

## 7. Strategies to Integrate the Variability in GastroPlus

**Figure 1:**
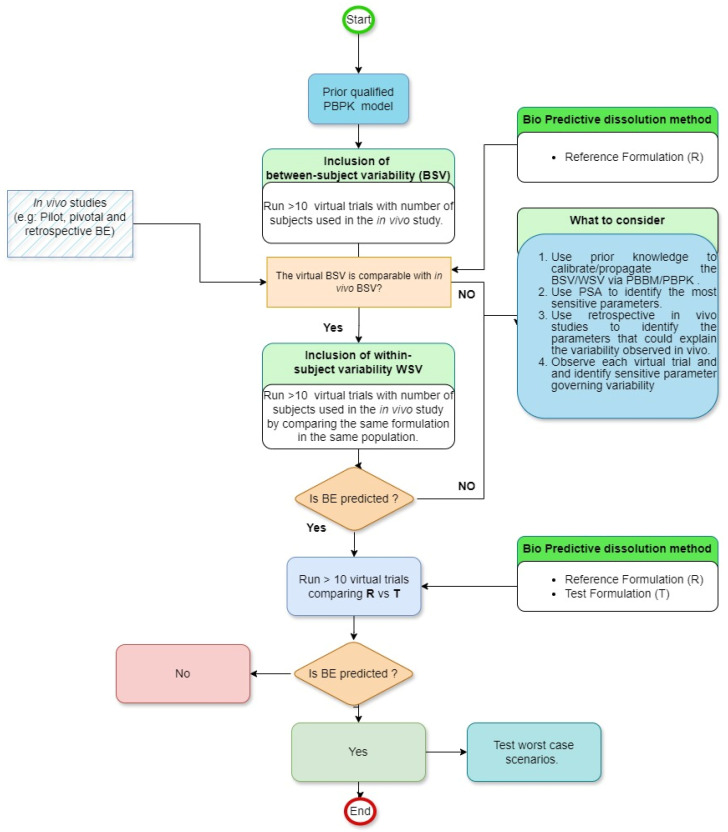
General workflow to perform VBE using GastroPlus.

To ensure accurate implementation of BSV and WSV, it is imperative to first develop a robust PBPK/PBBM model. This foundational step is paramount in determining whether drug absorption should be influenced by physiological factors or specific formulation parameters. Subsequently, the goal shifts to the development of a biopredictive dissolution method. Leveraging pilot, pivotal, or retrospective *in vivo* bioequivalence studies during this phase can be beneficial for both qualifying the PBPK model and establishing a biopredictive dissolution method. It is then essential to evaluate if the default BSV/WSV in the software can accurately forecast VBE. Different methodologies have been published as guidance on how to account for realistic BSV/WSV [16]. Figure 1 compiles the most recent advance in the area. This assessment involves determining if the virtual population has comparable BSV/WSV compared to that of observed in previous studies’ outcomes. If such a result is not achieved, the variability must undergo adjustments, as illustrated in Figure 1. The complexity of this process is compounded by the numerous physiological factors within the GI tract that influence oral drug absorption. Given the vastness of these factors, conducting direct experimental evaluations for all of them in the foreseeable future is challenging. Additionally, individual drugs and their formulations exhibit varied sensitivities to a spectrum of physiological parameters [37]. This means that the outcomes of sensitivity analyses are distinct and particular to each drug and its specific formulation. Finally, based on a parametric sensitivity analysis (PSA), the necessary sensitivity parameters should be identified and subsequently investigated for their impact on VBE. Once the origin and the value of BSV/WSV are identified, equal variability should be applied to investigate the BE between reference and test formulation. The goal of the virtual trial is to have the simulated BSV and WSV in PK parameters (e.g., C_max_ and AUC) close to BSV and WSV observed *in vivo*.

## 8. Real-World Application

Two case studies of developing a PBPK model and running VBE are presented below for IR and MR formulations, respectively.

### 8.1. Case Study 1

Molecule 1 is a BCS class II drug poorly soluble in aqueous media (<0.03 mg/mL) over the physiological pH range. The compound exhibited a dose-proportional increase in AUC and C_max_ after a single ascending dose. The molecule exhibited complete absorption under normal fasting conditions (fraction absorbed approximately 100% based on absolute bioavailability study). The purpose of the model was to develop a PBBM that could be used in the future to set dissolution specifications.

#### 8.1.1. Simulation Method

The simulations were conducted in GastroPlus^®^ v. 9.8 (Simulations Plus, Lancaster, CA, USA) using the default fasted Opt-logD SA/V v6.1 model and default absorption scaling factors. The API properties were gathered from literature (e.g., logP, pKa, blood to plasma partitioning ratio, binding to plasma proteins, etc.) or measured in-house (e.g., solubility at different pH, solubility in fasted state simulated intestinal fluid (FaSSIF) and simulated gastric fluid (SGF)). Human P_eff_ was calculated by GastroPlus internal conversion using P_app_ in Caco-2 cells.

Human PK parameters were estimated by fitting a three-compartment model to mean plasma concentration profiles after IV and oral capsule administration in PKPlus™. Capsule formulation was included to allow for proper elimination parameters estimation (sampling up to 840 h post-dose administration better captures long elimination half-life compared to 72 h post-dose sampling after IV administration).

The simulated PK profiles and parameters were compared to the observed human data obtained after IV and oral capsule administration. Percent prediction errors (%PE) were calculated for C_max_ and AUC as 100 × (observed value − predicted value)/(observed value).

For the evaluation of tablet formulations, a biorelevant dissolution method was used, consisting of 15 min in 300 mL SGF followed by 900 mL FaSSIF, pH 6.5. The *in vitro* dissolution data for test and reference tablet formulations from one pilot BE study with two test formulations and one reference formulation and one pivotal BE study with one test formulation and one reference formulation were entered in GastroPlus (*in vitro* dissolution vs. time profile files). Tripple Weibull parameters were fitted to dissolution data and saved as *in vivo* dissolution (*in vivo* controlled release vs. time profile files). The model for tablets was established, using all previously determined parameters, changing Dosage Form to CR: Dispersed, and using *in vivo* controlled release vs. time profile as dissolution input. The simulated PK profiles and parameters were compared to the observed human data obtained in pilot and pivotal BE studies, and %PE was calculated.

Then, 10 virtual populations with the same number of subjects as in the *in vivo* pivotal study and their plasma concentration profiles after administration of the reference formulation from the pivotal study were simulated. The number of subjects used in the population simulation was calculated based on prior clinical data. Total variability in the simulated C_max_ and AUC values (minimum and maximum) was compared to *in vivo* observed variability (minimum and maximum) in these parameters for the reference product. To match T_max_, the mean and %CV for the Tlag Weibull parameter were set to 0.5 h and 80%, respectively.

Then, crossover studies were simulated for the reference formulation. The population mean parameter and %CV in GastroPlus were used, except for the Tlag Weibull parameter, as described above. ISV was simulated using *in vivo* determined ISV on C_max_ and AUC.

Then, similarly, crossover studies were simulated for the test formulation from the pivotal BE study. Results were compared to *in vivo* observed test/reference C_max_ and AUC ratios.

#### 8.1.2. Results

**Figure 2:**
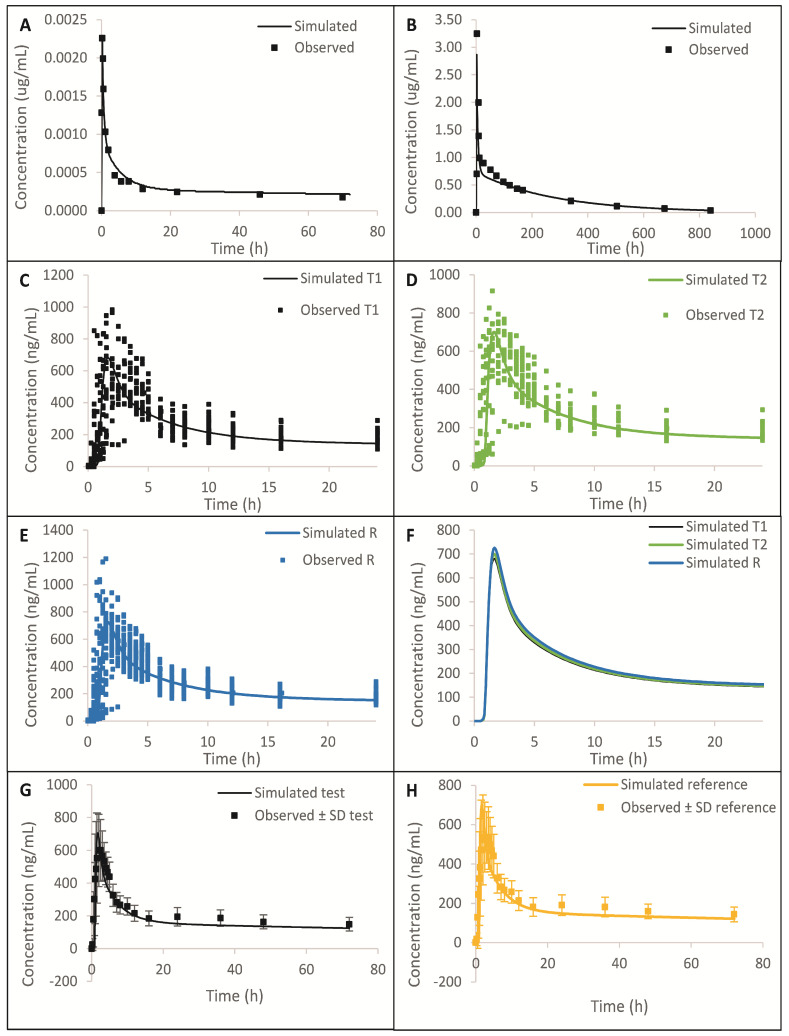
Simulated and observed plasma concentration profiles after IV administration (**A**), oral capsule administration (**B**), Test 1 tablet administration in pilot BE study (**C**), Test 2 tablet administration in pilot BE study (**D**), reference tablet administration in pilot BE study (**E**), Test 1, Test 2, and reference tablet administration in pilot BE study (**F**), test tablet administration in pivotal BE study (**G**), and reference tablet administration in pivotal BE study (**H**). Error bars in G and H are standard deviations of observed data.

The simulated plasma concentration vs. time profiles and comparison to the observed data are summarized in Figure 2 and Figure S1, which show that the model was able to predict observed data reasonably well after IV, higher strength oral capsule, and lower strength oral tablet administration. From Figure 2A,B and Figure S1, we can see that the distribution and elimination phases are well captured. A comparison of simulated and observed PK parameters and %PE are presented in Tables S1–S3. All %PEs were below 15%, which additionally confirms that the model adequately predicts *in vivo* data. In Table S4, we can see that the dissolution method is biopredictive, as it is able to adequately predict test/reference C_max_ and AUC ratios observed *in vivo*.

**Figure 3:**
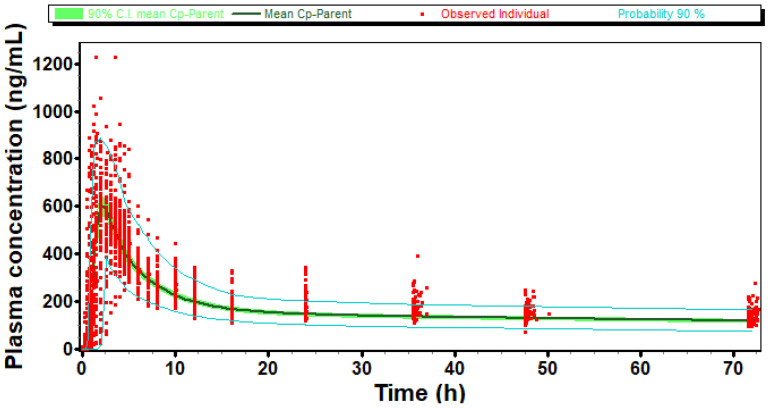
Simulated and observed individual plasma concentration profiles of Molecule 1 after administration of reference formulation from pivotal BE study.

A comparison is presented in Figure 3 of one simulated virtual population plasma concentration profile and *in vivo* observed individual plasma concentration profiles after reference administration in the pivotal study. We can observe that after adjustment of the T_lag_ Weibull parameter, simulated C_max_, AUC, and T_max_ variability of the reference formulation matched *in vivo* data.

All 10 virtual crossover BE trials confirmed that reference would be bioequivalent to itself in these trials.

**Figure 4:**
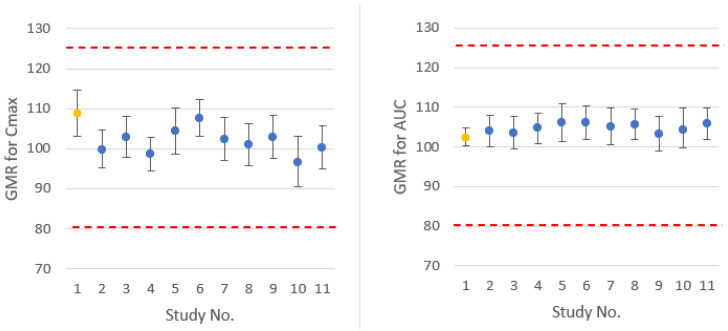
*In vivo* observed (yellow) and simulated in virtual trials (blue) point estimates and 90% confidence intervals of Molecule 1 for test/reference C_max_ and AUC geometric mean ratios. redlines: bioequivalence limits of 80–125%.

Virtual trials comparing test and reference formulations confirmed the BE of the formulations. Simulated test/reference C_max_ and AUC ratios and 90% confidence intervals are comparable to *in vivo* observed results, as presented in Figure 4.

### 8.2. Case Study 2

Molecule 2 belongs to BCS class I and is freely soluble throughout the GI pH conditions. It is formulated as an ER formulation. The compound exhibited a dose-proportional increase in AUC after both single and repeated dosing between dose ranges of 20–200 mg. Peak plasma concentrations were achieved 24 h after administration of ER formulation. The molecule is not metabolized significantly and becomes excreted unchanged in urine majorly. At a steady state, both IR and ER formulations are bioequivalent under the fasting condition. During the development, both fasting and fed bioequivalence studies have been conducted. Considering the ER product, three-time point-based specifications (1, 3, 8 h) have been recommended to cover the entire profile. At the 1 h time point, the specification is 10–30% (±10%), and at the 8 h time point, the specification is NLT 80%. However, at the 3 h time point, the specification is 33–58% (±12.5%) and exceeded the ±10% limit proposed by the regulatory agency. To support the dissolution specification at a 3 h time point, PBBM and simulation exercise along with VBE have been utilized.

#### 8.2.1. Simulation Method

The simulations were conducted in GastroPlus^®^ v 9.8.011 (Simulations Plus) using the default Opt-Log D SA/V V6.1 model and default absorption scaling factors. Physicochemical properties, such as solubility vs. pH data, log P, biorelevant solubility, and precipitation time, were gathered from the literature, and ADMET predictor values were used in the absence of literature information. Blood-to-plasma-concentration ratio and protein binding were obtained from the literature, and human effective permeability (P_eff_) was optimized based on data on *in vivo* plasma-concentration-time profiles.

Human elimination PK parameters were modeled using a two-compartment model. The IV and oral IR formulation data available in the literature were fitted using PKPlus^TM^, and elimination parameters were obtained after correcting them for bioavailability. The final obtained elimination parameters were further slightly optimized using an optimization module to have the best fit. Dosage forms such as IR tablet and CR integral tablet were utilized to describe the behavior of IR and CR formulations, respectively. For IR formulation, no dissolution data have been utilized, and simulations were based on particle size with the Johnson model. For CR formulation, dissolution data in the QC media has been utilized to simulate the *in vivo* profile. The dissolution data from QC media was proven to be bio-reflective, and these data were utilized in the modeling.

For all the single simulations, simulated PK profiles and parameters were compared to that of observed human data obtained after IR and MR formulations. % PEs were calculated for C_max_ and AUC as 100 × (observed value − predicted value)/(observed value).

The QC media consisted of 750 mL pH 7.5 phosphate buffer stirred with a paddle at 50 rpm. The QC media provided direction for *in vivo* results (e.g., lower dissolution of test product resulted in lower T/R ratios). However, it was observed that with direct input of QC media dissolution data, differences were observed with respect to observed vs. predicted T_max_. Direct input has resulted in shorter predicted T_max_ (e.g., 8–10 h), whereas observed T_max_ is significantly longer (e.g., 20–24 h). Due to differences in *in vitro* and possible *in vivo* dissolutions, *in vitro*–*in vivo* relationship (IVIVR) has been generated separately in the fasting and fed conditions using GastroPlus mechanistic deconvolution module to have appropriate time scaling on *in vitro* dissolution and to account for *in vivo* behavior. This exercise can result in matching predicted *in vivo* T_max_ with that of observed *in vivo* T_max_. After the generation of the IVIVR function, the *in vitro* dissolution has been converted to *in vivo* dissolution (*in vivo* controlled release vs. time profile file) and is directly used as input for pivotal reference and pivotal test formulations. Furthermore, the pharmacokinetics of pivotal test and reference formulations were predicted, and %PEs were calculated.

After that, virtual simulations have been conducted to develop a suitable population that can simulate *in vivo* behavior in fasting and fed conditions. For this purpose, 43 and 37 subjects (that were used in a pivotal clinical study) were used for fasting and fed conditions, respectively. Initial simulations were run with default variability using reference formulation, and further adjustments in the population parameters were performed to match clinically observed variability with that of the simulated variability. For the fasting condition, the default variability of all parameters was used except for clearance, where it was changed to 30% from the default value of 40%. Under fed conditions, the variability of clearance has been changed to 25%. Using these optimizations, the population appropriately covered the variability of all subjects under fasting and fed conditions.

Furthermore, VBE was conducted in the fasting and fed conditions, and it was observed that the clinical bioequivalence outcome in the fasting and fed conditions matched the simulated ratios. Furthermore, virtual dissolution profiles have been generated at the lower and upper specifications of the drug product. These dissolution profiles were converted into *in vivo* profiles using previously generated IVIVR, and VBE has been performed against pivotal test formulation using a previously validated population under fasting and fed conditions to justify dissolution specifications.

#### 8.2.2. Results

**Figure 5:**
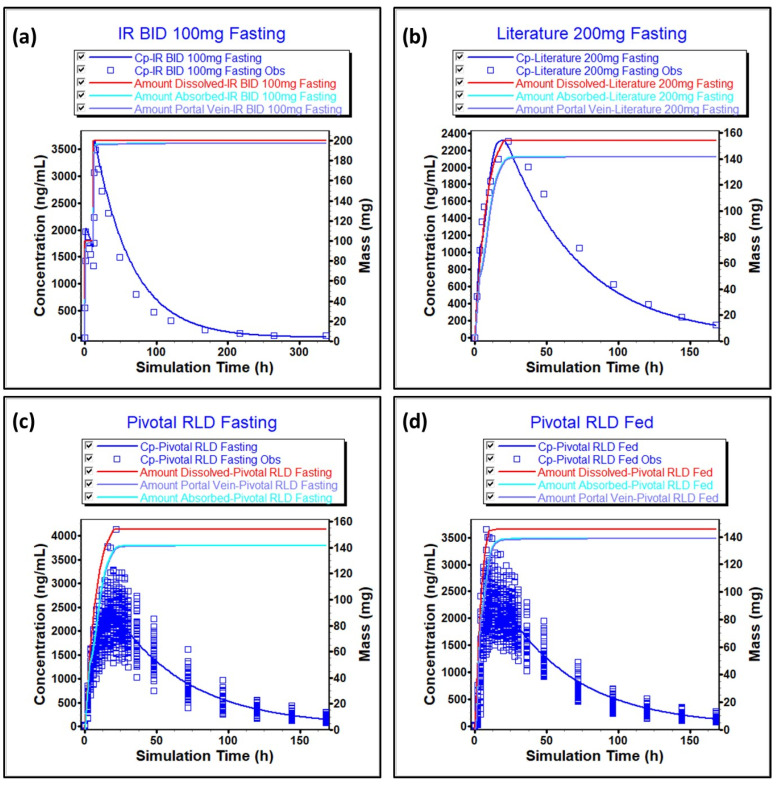
Model predictions for (**a**) literature IR BID 100 mg fasting and (**b**) literature ER 200 mg fasting; (**c**) in-house pivotal RLD fasting and (**d**) in-house pivotal RLD fed.

The simulated vs. observed plasma-concentration-time profile for literature-reported oral formulation is presented in Figure 5a. Furthermore, the model prediction against the literature, which reported 200 mg ER, is presented in Figure 5b. In both cases, the prediction errors are less than 15%, indicating the suitability of the model. The model also predicted the plasma-concentration-time profile of pivotal reference in both fasting and fed conditions using individual subjects’ data, as indicated in Figure 5c,d. The prediction errors in both cases are less than 15%, therefore indicating the suitability of the model.

**Figure 6:**
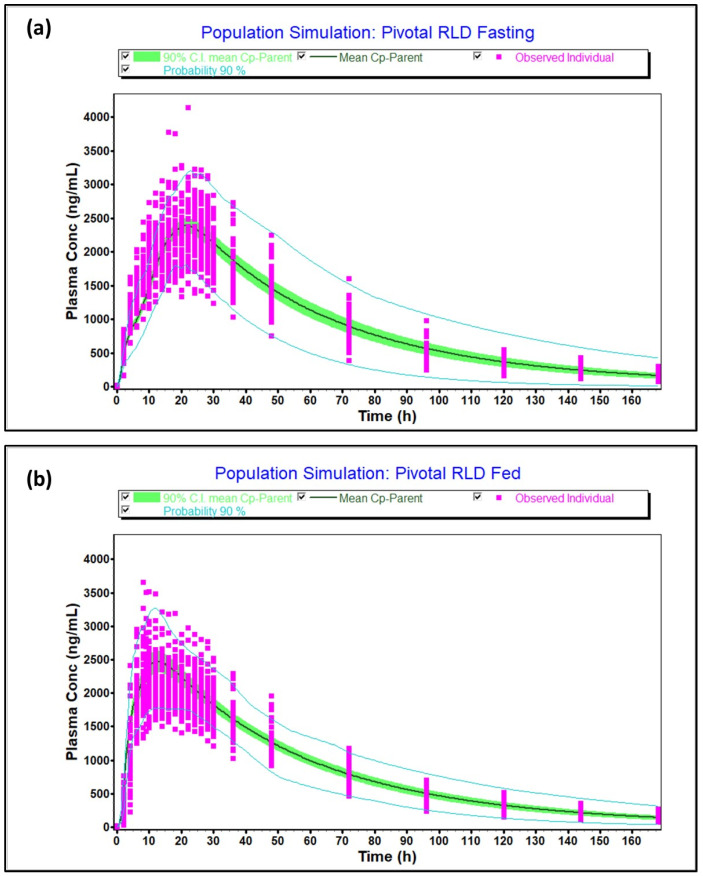
Virtual population for reference product in (**a**) fasting condition (**b**) fed condition.

The virtual optimized population under fasting and fed conditions captured the variability of the observed population as the 90% probability contours covered almost all the individual subjects, as shown in Figure 6a (fasting) and Figure 6b (fed). Moreover, the T/R ratio and 90% confidence intervals calculated between simulated and observed population were 102% [97.67–106.54%], 97.81% [91.51–104.55%] for C_max_ and AUCt in fasting and 106.2% [100.91–111.8%], 108.3% [102.98–113.93%] for C_max_ and AUCt in fed condition, respectively, and within 80.0–125.0%. Thus, the virtual population was found to be representative of the actual population studied in the clinic. Furthermore, the model predicted pivotal fasting and fed outcomes accurately, as indicated by Table S5.

The virtual dissolution profiles at the lower and upper dissolution specifications are provided in Table S6. The BE of lower and upper dissolution specifications against pivotal test formulation is provided in Table 1, and BE has been attained, thus justifying the dissolution specifications with PBBM modeling and the VBE approach.

## 9. Regulatory Experience on VBE

PBPK models and PBBM are now widely used for regulatory submissions to support various applications. VBE is used to assess bioequivalence between reference and test products to apply virtual population to various test variants in numerous cases (e.g., dissolution mismatch, biowaiver of lower strengths, justification of dissolution specifications, establishment of clinically relevant specifications, etc.) [16,17] Considering the importance of VBE, regulatory agencies such as U.S. FDA and EMA have come up with respective regulatory guidance [38,39,40]. U.S. FDA PBBM guidance [39] mentions the utility of VBE to predict the effect of variations from the CMAs, CPPs, and CQAs on drug exposure toward the establishment of a safe space via either IVIVCs or *in vitro*–*in vivo* relationships. This guidance also details the utility of VBE in establishing a dissolution safe space to link *in vitro* dissolution with *in vivo* behavior, with further emphasis on the number of subjects and variability considerations for designing VBE studies. EMA PBPK guidance [40] mentions the selection of suitable virtual populations that are representative of the target population for various applications.

Regulatory agencies such as the U.S. FDA have encouraged the utilization of PBPK modeling and VBE at both new drug and generic product development through various conferences over the past few years. During these conferences, VBE was one of the main topics discussed. Fang Wu [41] talked about PBPK modeling and VBE in supporting regulatory decision-making. It was detailed that VBE has been a part of model validation and application and occupies significance as it incorporates intended variability into simulations. Such simulations were found to have an impact on regulatory decision-making through properly developed and validated models. All the case examples indicated the importance of VBE in decision-making through the selection of the appropriate number of subjects. Shoyaib [42] talked about the utility of VBE in predicting fed bioequivalence for new drugs and generic products. It was indicated that PBPK models are used to study drug-food interactions due to their ability to simulate food intake mechanistically. Furthermore, food impact on BE can be determined via VBE simulations, and the same can be demonstrated using acyclovir as a case example. Furthermore, during the 2021 U.S. FDA CRCG workshop on the regulatory utility of PBPK modeling to support alternative bioequivalence and risk assessment [43], VBE approaches were discussed in detail. It was discussed that VBE can be utilized to support safe spaces of critical quality attributes. This concept is also evident from the U.S. FDA’s recent presentations on critical bioavailability attributes (CBAs), which are defined as any formulation variable that can impact the bioavailability or bioequivalence of the formulation. Recently, Ahmed et al. [44] proposed CBA’s evaluation and biopharmaceutics risk assessment workflow for generic regulatory submissions. This publication suggests the use of PBBM for the evaluation of risk assessment and CBA evaluation that utilizes VBE to understand formulation attributes that can result in bio-inequivalence against reference products. Furthermore, Kollipara et al. [45] talked about the utilization of PBBM in the waiver of fed bioequivalence studies. In this publication, the possibility of fed bioequivalence study waivers utilizing PBBM with respect to BCS class, formulation, and type of food effect was discussed. These publications summarized that fed bioequivalence waivers with PBBM are possible with the help of biopredictive media development and validation. Additionally, a positive food effect arising from solubility or dissolution rate enhancement of poorly soluble molecules (BCS II and IV) is possible with PBBM. However, to simulate negative food effects, the exact mechanism underlying the impact of food needs to be understood and incorporated into the model. However, overall, VBE has been recognized as a potential area by regulatory agencies in biopharmaceutical risk assessment, bioavailability, and bioequivalence evaluations.

In our experience of submission of PBBM and VBE to regulatory agencies as a part of generic regulatory submissions, a few queries have been received. Often, agency requests to validate the model against failed bioequivalence studies to demonstrate the robustness of the model. Prediction of bio-inequivalence along with the T/R ratio and 90% CI can enhance the capability of the model. Although the number of VBE trials to be conducted for PBBM is not specified, typically, more than 10 VBE with different populations can be performed to further demonstrate the robustness of the model. Furthermore, agencies also request an evaluation of CBA’s using PBBM for regulatory submissions. The integration of CBA in the model coupled with VBE can identify the boundaries of formulation variables and help establish a safe space, therefore embedding clinical quality into product development. An underlying assumption in successful PBBM is that the dissolution is biopredictive. Demonstrating the discriminatory power of dissolution through *in vitro* and *in vivo* modeling can help reject batches that can exhibit potential bio-inequivalence, and VBE can be of significant aid in such scenarios. Furthermore, VBE is an indispensable tool for the demonstration of biowaivers and f2 mismatch and for evaluating gender impact. It can definitely enable both generic and new drug submissions.

## 10. Conclusions and Future Perspectives

In this manuscript, we have reviewed the current understanding of VBE, including sources of *in vivo* variability and the incorporation of these in VBE trails within GastroPlus. As shown by two case examples, PBBM with VBE can be used for regulatory purposes, laying out options for waiving unnecessary bioequivalence studies. We have proposed a state-of-the-art workflow for performing effective VBE using GastroPlus. Although we have demonstrated the utility of this workflow using only two case examples, we will be validating this workflow further using multiple other drug products in the future. The observations from this exercise will be shared in our future publications. This manuscript provides a workflow for GastroPlus-based VBE. Similar concepts also can be extrapolated to other software tools such as SimCyp and PKSIM.

In the foreseeable future, *in vivo*, bioequivalence studies will still be needed to put or keep generic drug products on the market, especially in view of the recently published ICH M13A draft guideline [46]. According to this guideline, a rationale should be provided for the selection of the type of BE study(ies) (fasting or fed or both) and meal type, e.g., fat and calorie content, based on the understanding of the comparator product and the test product. The rationale can be supported by modeling, e.g., appropriately validated/qualified PBPK modeling or semi-mechanistic absorption models, which portrays possibilities for PBBM and VBE studies being used for regulatory purposes as well as internal decision-making.

## Figures and Tables

**Table 1:** Fasting and Fed state virtual simulations between proposed lower and upper specifications (as test) against pivotal test (as reference).

BE Assessment	C_max_ T/R (90% CI)	AUCinf T/R (90% CI)	AUCt T/R (90% CI)
Fasting State			
Lower spec vs. Test	90.8 (87.2–94.5)	87.6 (80.4–95.6)	87.4 (81–94.4)
Upper spec vs. Test	107.0 (102.6–111.7)	108.6 (99.9–118.1)	108.9 (101.2–117.2)
Fed State			
Lower spec vs. Test	96.8 (91.1–102.7)	96.1 (89.7–102.9)	95.8 (90.1–101.9)
Upper spec vs. Test	105.3 (99.4–111.5)	102.3 (96–109.1)	102.5 (96.9–108.4)

## Data Availability

No new data were created or analyzed in this study. Data sharing is not applicable to this article.

## Supplementary Materials

The following supporting information can be downloaded at: https://www.mdpi.com/article/10.3390/ph17070876/s1, Table S1: Simulated and observed PK parameters and calculated %PE of Molecule 1 for IV and oral capsule administration; Table S2: Simulated and observed PK parameters and calculated %PE of Molecule 1 for two test formulations and one reference formulation from pilot BE study; Table S3: Simulated and observed PK parameters and calculated %PE of Molecule 1 for one test and one reference formulation from pivotal BE study; Table S4: Simulated and observed test/reference C_max_ and AUC ratios and calculated %PE of Molecule 1; Table S5. Population predicted and observed T/R ratios for pivotal RLD and Test in fasting and fed conditions for Molecule 2. Table S6: Upper and lower dissolution specifications dissolution profiles against the pivotal test batch for Molecule 2. Figure S1: Simulated and observed plasma concentration profiles (log scale) after IV administration (A), oral capsule administration (B), test 1 tablet administration in pilot BE study (C), test 2 tablet administration in pilot BE study (D), reference tablet administration in pilot BE study (E), test 1, test 2, and reference tablet administration in pilot BE study (F), test tablet administration in pivotal BE study (G), and reference tablet administration in pivotal BE study (H). Error bars in G and H are standard deviations of observed data.

## Abbreviations

ADME—Absorption Distribution Metabolism Elimination; AIC—Akaike Information Criterion; API—Active pharmaceutical gradient, AUC—Area under the curve; BCS—Biopharmaceutics classification system; BSV—Between-subject variability; CI—Confidence interval; CL—Clearance; C_max_-Maximum plasma concentration; CV—Coefficient of variation; DDI—Drug-drug interactions; DR—Delayed release; EMA—European Medicines Agency; ER—Extended release; F2-Similarity factor; FaSSIF—Fasted state simulated intestinal fluid; GI—Gastrointestinal; IOV—Inter-occasion variability; IR—Immediate release; ISV—Intra-subject variability; IV—Intravenous; IVIVR—*In vitro*–*in vivo* relationship; Km-Michaelis-Menten constant; MRT—Mean residence time; PBBM—Physiologically based biopharmaceutics modeling; PBPK—Physiologically based pharmacokinetic modeling; SGF—Simulated Gastric Fluid; T_max_—Time at which maximum plasma concentration is reached; U.S. FDA—United States Food and Drug Administration; VBE—Virtual Bioequivalence; Vmax-Maximum velocity of reaction; WSV—Within-subject variability
