# Supplementary material for: Advancing Virtual Bioequivalence for Orally Administered Drug Products: Methodology, Real-World Applications and Future Outlook
Source: Pharmaceuticals (Basel). 2024 Jul 3;17(7):876. doi: 10.3390/ph17070876 (PMC11279853; doi:10.3390/ph17070876)
Supplement: Supplementary file 1 [file pharmaceuticals-17-00876-s001.zip › pharmaceuticals-3006718-supplementary.pdf]

Advancing Virtual Bioequivalence for Orally Administered Drug Products: Best Practices and Real-World Applications

Sivacharan Kollipara [1], Frederico Severino Martins [2], Rebeka Jereb [3], Dejan Krajcar [3] and Tausif Ahmed [1]*

[1] Biopharmaceutics Group, Global Clinical Management, Dr. Reddy's Laboratories Ltd., Integrated Product Development Organization (IPDO), Bachupally, Medchal Malkajgiri District, Hyderabad-500 090, Telangana, India.
[2] Simulations Plus, Inc., Lancaster, CA, USA.
[3] Clinical Pharmacology and Modeling & Simulation, Sandoz Global Development, Lek d. d., Verovškova ulica 57, SI – 1526 Ljubljana, Slovenia.
* Correspondence: tausifahmed@drreddys.com, Tel: +91-40-4434200

Supplementary Materials.

Table S1: Simulated and observed PK parameters and calculated %PE of Molecule 1 for IV and oral capsule administration; Table S2: Simulated and observed PK parameters and calculated %PE of Molecule 1 for two test and one reference formulation from pilot BE study; Table S3: Simulated and observed PK parameters and calculated %PE of Molecule 1 for one test and one reference formulation from pivotal BE study; Table S4: Simulated and observed test/reference C_{max} and AUC ratios and calculated %PE of Molecule 1; Table S5. Population predicted and observed T/R ratios for pivotal RLD & Test in fasting & fed conditions for Molecule 2. Table S6: Upper and lower dissolution specifications dissolution profiles against the pivotal test batch for Molecule 2; Figure S1: Simulated and observed plasma concentration profiles (log scale) after IV administration (A), oral capsule administration (B), test 1 tablet administration in pilot BE study (C), test 2 tablet administration in pilot BE study (D), reference tablet administration in pilot BE study (E), test 1, test 2, and reference tablet administration in pilot BE study (F), test tablet administration in pivotal BE study (G), and reference tablet administration in pivotal BE study (H). Error bars in G and H are standard deviations of observed data.

Table S1. Simulated and observed PK parameters and calculated %PE of Molecule 1 for IV and oral capsule administration.

PK parameter	Metric	IV	Oral capsule
**C_{max} (μg/mL)**	Observed	0.0024	3.4
	Predicted	0.0023	2.9
	%PE	**2.7**	**14.5**
**AUC_t (μg*h/mL)**	Observed	0.0699	207
	Predicted	0.0752	196
	%PE	**-7.6**	**5.1**
**T_{max} (h)**	Observed	0.23	1.5
	Predicted	0.25	1.9

Table S2. Simulated and observed PK parameters and calculated %PE of Molecule 1 for two test and one reference formulation from pilot BE study.

PK parameter	Metric	Test 1	Test 2	Reference
**C_{max} (μg/mL)**	Observed	664.5	672.4	702.9
	Predicted	680.8	700.1	725.7
	%PE	**-2.5**	**-4.1**	**-3.2**
**AUC_t (μg*h/mL)**	Observed	13434	13655	13566
	Predicted	11760	12010	12500
	%PE	**12.5**	**12.0**	**7.9**
**T_{max} (h)**	Observed	2.0	2.5	2.0
	Predicted	1.7	1.7	1.7

Table S3. Simulated and observed PK parameters and calculated %PE of Molecule 1 for one test and one reference formulation from pivotal BE study.

PK parameter	Metric	Test 1	Reference
**C_{max} (μg/mL)**	Observed	733.9	674.5
	Predicted	705.9	700.6
	%PE	**3.8**	**-3.9**
**AUC_t (μg*h/mL)**	Observed	14241	13901
	Predicted	12410	12080
	%PE	**12.9**	**13.1**
**T_{max} (h)**	Observed	2.5	2.5
	Predicted	1.8	1.7

Table S4. Simulated and observed test/reference C_{max} and AUC ratios and calculated %PE of Molecule 1.

PK parameter	Metric	Pilot study	Pivotal study

		Test 1/Reference	Test 2/Reference	Test/Reference
**C_{max} ratio**	Observed	91.38%	95.66%	108.81%
	Predicted	93.81%	96.48%	100.75%
	%PE	**-2.7**	**-0.9**	**-8.0**
AUC ratio	Observed	93.74	100.66%	102.45%
	Predicted	94.08	96.08%	102.73%
	%PE	**-0.4**	**4.5%**	**0.3**

Table S5. Population predicted and observed T/R ratios for pivotal RLD & Test in fasting & fed conditions for Molecule 2.

**PK parameter**	**Observed**		**Predicted**		**Prediction error (%)**
**Fasting condition**					
	**T/R ratio**	**90% confidence interval**	**T/R ratio**	**90% confidence interval**	
C_{max}	86.57	82.22 - 91.15	89.96	86.35-93.73	3.92
AUCinf	90.86	86.68 - 95.24	91.06	84.26-98.42	0.22
AUCt	91.29	87.14 - 95.63	90.95	83.31-99.29	0.37
**Fed condition**					
Cmax	110.05	104.08 - 116.36	96.68	91.19-102.5	12.15
AUCinf	105.34	101.31 - 109.53	99.12	93.63-104.93	5.90
AUCt	105.13	101.38 - 109.02	99.23	93.03-105.85	5.61

Table S6: Upper and lower dissolution specifications dissolution profiles against the pivotal test batch for Molecule 2.

**Time (h)**	**Pivotal Test**	**Proposed specification**	**Lower specification**	**Upper specification**
0	0		0	0
0.5	10		0	24
1	17	10-30%	6	31
2	31		20	45
3	44	33-58%	33	58
4	56		45	70
6	76		65	90
8	89	NLT 80%	80	100
10	96		87	100

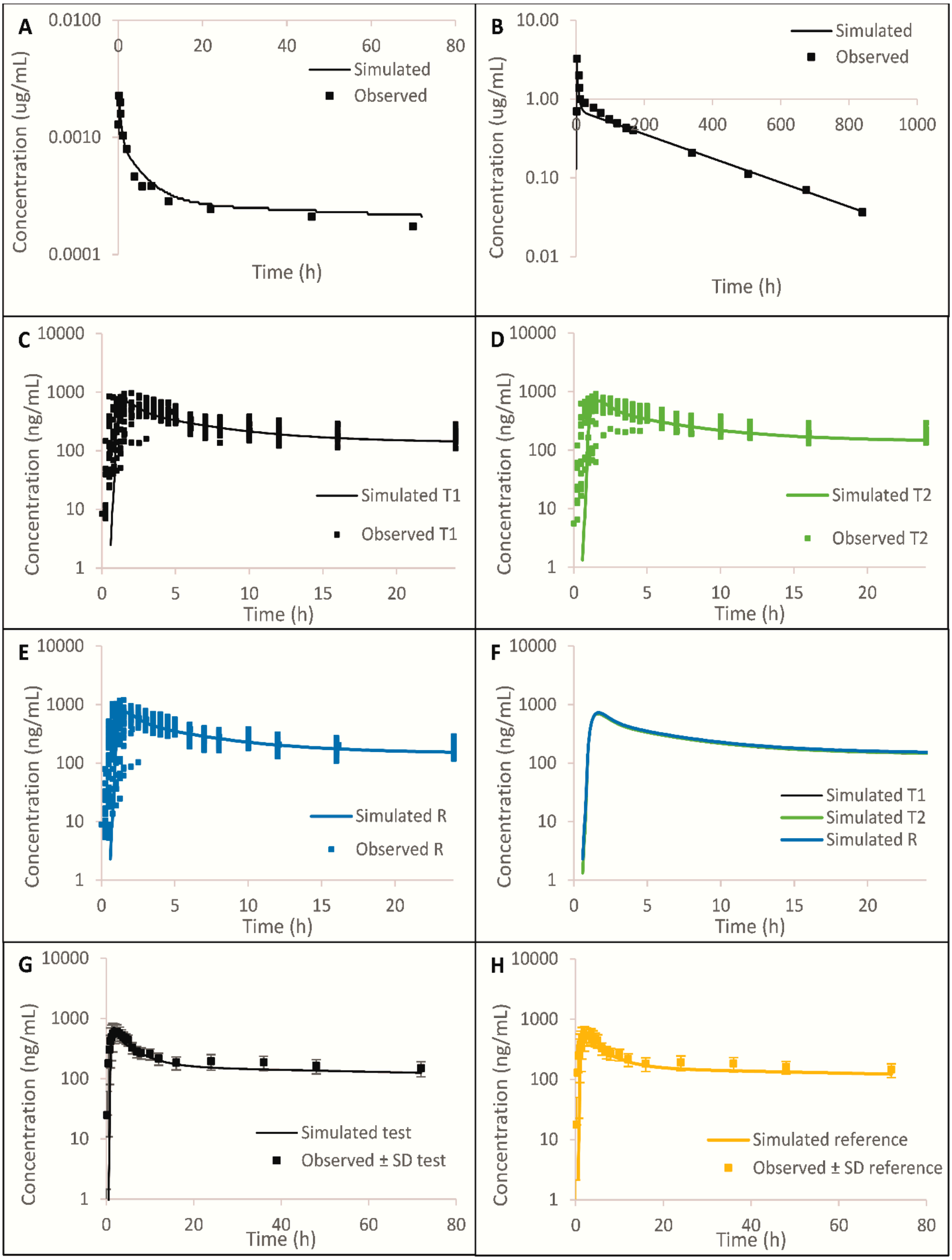

Figure S1. Simulated and observed plasma concentration profiles (log scale) after IV administration (A), oral capsule administration (B), test 1 tablet administration in pilot BE study (C), test 2 tablet administration in pilot BE study (D), reference tablet administration in pilot BE study (E), test 1, test 2, and reference tablet administration in pilot BE study

(F), test tablet administration in pivotal BE study (G), and reference tablet administration in pivotal BE study (H). Error bars in G and H are standard deviations of observed data.
